# Neuropsychiatric Symptoms of Dementia: Consent, Quality of Life, and Dignity

**DOI:** 10.1155/2013/230134

**Published:** 2013-06-20

**Authors:** Michael J. Passmore

**Affiliations:** Department of Psychiatry, Geriatric Psychiatry Program, University of British Columbia, c/o Mount Saint Joseph Hospital, Ward 1 South, 3080 Prince Edward Street, Vancouver, BC, Canada V5T 3N4

## Abstract

Degenerative forms of dementia are progressive, incurable, fatal, and likely to cause suffering in conjunction with personal incapacity. Timely diagnostic disclosure and counseling can facilitate important advance care planning. The risk of harm associated with neuropsychiatric symptoms (NPS) of dementia often has to be balanced against the risk of harm associated with medication management of NPS. A palliative care framework can help preserve autonomy, quality of life, comfort, and dignity for patients with NPS.

This is not a topic for polite conversation. Then again, dementia is not a polite illness. The ugly reality is that dementia often manifests as a relentless and cruel assault on personhood, comfort, and dignity. Up to 90% of people with dementia will suffer behavioral and/or psychological symptoms (BPSD) [1]. Otherwise known as neuropsychiatric symptoms (NPS) of dementia; these horrible problems metastasize as dementia siphons away control over thoughts and actions, control that we take for granted every waking second of every day. A spouse no longer recognized becomes an intruder and is then attacked in terror. A lifelong neighborhood becomes foreign, and is then a confusing labyrinth. Intractable tearfulness, sobbing, and pleading for help when no identifiable threat actually exists. Struggling against a padded restraint, smearing excrement, and wailing.

Although such graphic experiences are not universal among dementia sufferers, NPS involving rampant psychosis, anxiety, and agitation can present a haunting affront to personhood, dignity, and quality of life. Furthermore, these types of NPS often jeopardize the safety of patients and caregivers. Therein lay the risks of harm often associated with unabated NPS. Yet when it comes to considering the risk-benefit potential of NPS treatment options, most patients in this state are not capable of providing informed consent, and very few have indicated ahead of time how they might wish to be treated under such circumstances [2]. For guidance, clinicians may turn to a substitute decision-maker and indeed turn inwards in an empathic attempt to relieve suffering. A clinician might ask “How can I help my patient be less paranoid of his loved ones, less prone to becoming lost outside on a cold winter night, less likely to fight with caregivers who work to help him maintain his personal hygiene when he no longer can? If there are no definitive treatment options, should I prescribe a medication to help calm him? What if the medication has side-effects? He might be more sedate, perhaps even requiring a wheelchair most of the time. Would he consider these side effects to be acceptable? Would it matter to him if he knew that he would eventually lose the ability to walk, talk, swallow and remain alert as a result of end-stage dementia anyway? What if the medication increased the risk of a very serious problem like pneumonia, a stroke or a fatal arrhythmia? How much of an increased risk of stroke or death would be acceptable to him? Would he take the medication if it caused a 50% annual risk of stroke or death? Would he even care about an increased risk of death? What if the medication eventually caused a stroke that left him alive but with even more suffering than before? Does he consider the quantity of his life to be more important than the quality of his life? What aspects of life does he value? Are there aspects of life that he absolutely requires? Are there any foreseeable circumstances under which he would not want to live?”

Aside from the bleak fact that it is almost always incurable and fatal, the truly vexing thing about dementia is that it is very difficult to predict how and when things will worsen. Thus, there is no pressing impetus to ask cognizant patients these tough questions ahead of time. Although there are general patterns of decline corresponding to the natural history of specific dementia types, clinicians cannot rely on biomarkers (yet) or tissue grading/staging to guide diagnosis, targeted management, or prognosis. On the other hand, clinicians can at least inform patients and their family member(s) of two important points: (i) that dementia is not a normal part of aging and (ii) that dementia is very likely to worsen and eventually lead to death. It seems important that patients understand this early in the course of illness because they can then enter into discussions around goals of care and plan ahead for a time when they may be in a life-limiting state of suffering and simultaneously not capable of making decisions for themselves. Decisions like what kind of medication side effects/risks would be acceptable for debilitating NPS. Decisions like if and when he should move out of the home he built 48 years ago and into a 500 square foot room shared with three other bed-bound incontinent people separated by beige curtains.

While diagnostic disclosure of dementia has been associated with improved quality of life [3] and is recommended in treatment guidelines [4], the discussion remains challenging. Reasons for deferring or avoiding disclosure include diagnostic uncertainty, fear of stigmatization, cultural preferences, and competing expectations between patients and their companion(s) [5–8]. Despite these challenges, a recent systematic review found that patients want to be included in the diagnostic disclosure so that they can come to terms with the illness, try to maintain normality, and receive ongoing counseling [9]. Not only are catastrophic reactions unlikely, in fact, anxiety usually decreases following a diagnostic discussion [10]. Components of a practical patient-centered dementia disclosure include diagnostic education, discussion of management goals, and provision of realistic hope with a focus on nonabandonment [11].

In dementia, as with other terminal illnesses, the diagnostic discussion can be conceived as one of those truly sacred clinician-patient interactions. As such, a blunt, rushed, or forced diagnostic disclosure can inadvertently betray the very patient autonomy it may be seeking to uphold [12]. This might occur in cases when personal, family, or cultural values entail preference for less direct communication of technical medical matters or even complete deference to the judgment of the physician in terms of directing care. Accordingly, respect for autonomy does not simply involve labeling patients and then leaving them with à la carte management options to pick and choose from. Instead, clinicians are invited to align the approach to dementia diagnosis and management with an intuitive and empathic understanding of patients' values and preferences so that patient-centered goals of care can be gently identified and then ardently pursued.

It is also important to involve family caregivers in the diagnostic counseling process well before patients are hospitalized with advanced dementia and NPS. In hospital, my colleagues and I often hear from patients' family members that neither they nor the patient were ever told that dementia would most likely lead to death. The family members then find themselves grieving just as they hand over care of a significantly impaired loved one to a group of complete strangers. Consequently, sparks of denial and anger ignite a tinderbox of resentment, guilt, and exhaustion built up over years of care at home. A trusting therapeutic relationship is difficult to establish under such stressful conditions. Our patients, if aware, would surely be disheartened to find their family members and clinicians struggling against one another instead of struggling together with them against their illness.

Indeed, the nature of this struggle merits careful consideration. Much of the recent literature on medication management of NPS has focused on the risk of morbidity and mortality associated with antipsychotics [13] and other psychotropics [14]. While knowledge of this harm evidence is important for a patient/substitute decision maker during informed consent discussions, it can also be misinterpreted as a reason to shun medications that may enhance a suffering patient's quality of life. Quality of life that many, if not most, people would wish to have optimized in the face of an incurable and fatal illness like dementia that threatens self-control and dignity. In addition to the literature indicating a clinically significant risk of harm with antipsychotic use for management of NPS [15], there is evidence that this risk of harm outweighs the effectiveness of these medications in outpatients with mild to moderate NPS [16]. On the other hand, when the risk of suffering due to under-treated NPS is factored into the equation, there can be a utility advantage to judicious use of antipsychotic medication after less risky management options have been exhausted [17]. A recent study in which quality of life was a primary outcome measure found that NPS consistently detracted from quality of life, whereas antipsychotic use did not [18]. Furthermore, emerging evidence is confirming what would intuitively be feared as a result of NPS under treatment. When previously helpful medications are withdrawn, NPS can resurface and quality of life can suffer [19–21]. Quality of life must also be protected by our ongoing efforts to identify and treat common problems contributing to NPS, such as delirium and pain [22]. Given the daunting scope of these problems, resource allocation must allow for the development of care environments that are conducive to meaningful activity appropriate for a given patient's functional capacity throughout the course of illness [23].

In view of the complexity and controversy surrounding NPS management, it can be helpful to adopt a framework that guides clinical decision making. A framework that rests on the premise that dementia is almost always progressive, incurable, fatal, and likely to cause suffering in conjunction with personal incapacity. Palliative care is often misunderstood as limited to patient care in the final hours, days, or weeks of life. Accordingly, palliative care is often not considered to be appropriate for patients with dementia until they are at the very end stage of impairment [25]. In this sense, palliative care becomes confused with end-of-life care and terminal care when, in fact, all three approaches to care coexist on a continuum (Figure 1) [24]. Once this is appreciated, it becomes clear that early adoption of a palliative care approach in dementia is worthy of consideration. A palliative approach to dementia care seeks to preserve quality of life, comfort, and dignity by developing an individualized care plan involving meaningful activity, appropriate medical care, and treatment of behavioral symptoms [26, 27]. Accordingly, a palliative approach does not necessarily mean defaulting to “conservative” care, whereby quality of life is prioritized over quantity of life. Instead, the goal is to help patients and their family members come to terms with the diagnosis while guiding consent decisions and aligning illness management with the patient's core values. The concept of health promotion as it relates to palliative care has previously been proposed as an important method of steering public health policy in more participatory and collaborative directions [28].

Some might criticize the early adoption of palliative care in dementia, citing therapeutic nihilism, or “giving up hope” when, in fact, recognition of palliative care needs can maintain the hope that a patient will have the opportunity to guide his/her care in accordance with principles and values that are near and dear to his/her heart. Perhaps some clinicians tend to defer palliative care considerations because of unconscious denial or projection of their own anxiety regarding vulnerability, suffering, and death. Recognition of any such personal bias can be helpful when counseling patients and their family members. Counseling can support patients in part by providing strategies for coping with the uncomfortable reality of what lies ahead. Such strategies can include referral to community peer support services such as The Alzheimer Society of Canada via the First Link program (http://www.alzheimer.ca/). Discussion of advance directives can give patients the opportunity to think about what kind of care they would want for themselves in the event that they are no longer capable of directing their own care. In British Columbia, the My Voice document [29] can help patients develop and implement advance directives in accordance with recent legislative changes that accommodate the need for such planning. Another useful advance care planning tool is the Five Wishes program provided by the organization Aging With Dignity (http://www.agingwithdignity.org/five-wishes.php).

Even more reason for hope is that the extensive ongoing research on dementia will continue to improve our ability to diagnose, manage, and prognosticate. In the meantime, despite all of the inherent complexity, controversy, and uncertainty, much can be done for our patients based on what is known. Degenerative forms of dementia are progressive, incurable, fatal, and likely to cause suffering in conjunction with personal incapacity. Open consideration of this to the extent felt necessary for any given patient can provide an opportunity for patients to guide their future care as much or as little as they wish.

## Figures and Tables

**Figure 1:**
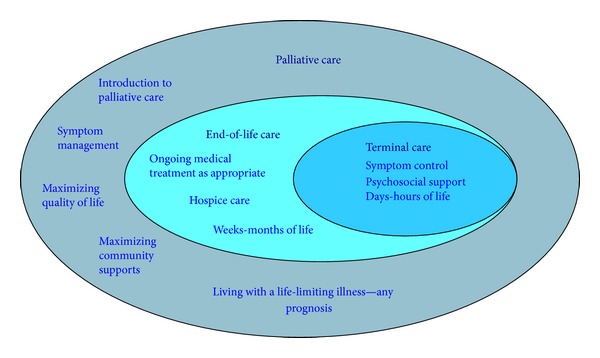
The palliative care continuum [24].
